# Population‐based study of anastomotic stricture rates after minimally invasive and open oesophagectomy for cancer

**DOI:** 10.1002/bjs5.50176

**Published:** 2019-06-10

**Authors:** O. Helminen, V. Kytö, J. H. Kauppila, J. Gunn, J. Lagergren, E. Sihvo

**Affiliations:** ^1^ Department of Surgery Central Finland Central Hospital Jyväskylä Finland; ^2^ Cancer and Translational Medicine Research Unit, Medical Research Centre Oulu University of Oulu Oulu Finland; ^3^ Department of Surgery Oulu University Hospital Oulu Finland; ^4^ Heart Centre Turku University Hospital Turku Finland; ^5^ Research Centre of Applied and Preventive Cardiovascular Medicine University of Turku Turku Finland; ^6^ Department of Surgery, Faculty of Medicine University of Turku Turku Finland; ^7^ Upper Gastrointestinal Surgery, Department of Molecular Medicine and Surgery, Karolinska Institutet Karolinska University Hospital Stockholm Sweden; ^8^ School of Cancer and Pharmaceutical Sciences King's College London London UK; ^9^ Guy's and St Thomas' NHS Foundation Trust London UK

## Abstract

**Background:**

The population‐based incidence of anastomotic stricture after minimally invasive oesophagectomy (MIO) and open oesophagectomy (OO) is not known. The aim of this study was to compare rates of anastomotic stricture requiring dilatation after the two approaches in an unselected cohort using nationwide data from Finland and Sweden.

**Methods:**

All patients who had MIO or OO for oesophageal cancer between 2007 and 2014 were identified from nationwide registries in Finland and Sweden. Outcomes were the overall rate of anastomotic stricture and need for single or repeated (3 or more) dilatations for stricture within the first year after surgery. Multivariable Cox regression provided hazard ratios (HRs) with 95 per cent confidence intervals, adjusted for age, sex, co‐morbidity, histology, stage, year, country, hospital volume, length of hospital stay and readmissions.

**Results:**

Some 239 patients underwent MIO and 1430 had an open procedure. The incidence of strictures requiring one dilatation was 16·7 per cent, and that for strictures requiring three or more dilatations was 6·6 per cent. The HR for strictures requiring one dilatation was not increased after MIO compared with that after OO (HR 1·19, 95 per cent c.i. 0·66 to 2·12), but was threefold higher for repeated dilatations (HR 3·25, 1·43 to 7·36). Of 18 strictures following MIO, 14 (78 per cent) occurred during the first 2 years after initiating this approach.

**Conclusion:**

The need for endoscopic anastomotic dilatation after oesophagectomy was common, and the need for repeated dilatation was higher after MIO than following OO. The increased risk after MIO may reflect a learning curve.

## Abstract (es)

**Antecedentes:**

Se desconoce la incidencia poblacional de estenosis anastomóticas tras esofaguectomía mínimamente invasiva (*minimally invasive oesophagectomy*, MIO) y esofaguectomía abierta (*open oesophagectomy*, OO). El objetivo de este estudio fue comparar las tasas de estenosis anastomótica que precisan dilataciones después de los dos abordajes de esofaguectomía en una cohorte no seleccionada utilizando los datos poblacionales de Finlandia y Suecia.

**Métodos:**

Todos los pacientes sometidos a MIO (*n* = 239) o OO (*n* = 1430) por cáncer de esófago entre 2007 y 2014 fueron identificados a partir de los registros nacionales en Finlandia y Suecia. Las variables de resultados fueron la incidencia global de estenosis anastomótica y la necesidad de una sola dilatación o dilataciones repetidas (≥ 3) para la estenosis durante el primer año de la cirugía. La regresión multivariable de Cox proporcionó los cocientes de riesgos instantáneos (*hazard ratios*, HRs) con los i.c. del 95% ajustados por edad, sexo, comorbilidad, histología, estadio, año, país, volumen del hospital, duración de la estancia hospitalaria y reingresos.

**Resultados:**

La incidencia de estenosis que precisaron una dilatación fue del 16,7%, y del 6,6% para estenosis que precisaron ≥ 3 dilataciones. El HR de estenosis que requirieron una dilatación no se incrementó tras MIO en comparación con OO (HR 1,19, i.c. del 95% 0,66‐2,12), pero fue 3 veces más para dilataciones repetidas (≥ 3) (HR 3,25, i.c. del 95% 1,43‐7,36). De las 18 estenosis tras MIO, 14 (78%) ocurrieron durante los primeros dos años en los que se inició este abordaje.

**Conclusión:**

La necesidad de dilatación endoscópica de la anastomosis tras esofaguectomía fue frecuente y la necesidad de dilataciones repetidas fue más alta tras MIO en comparación con OO. El riesgo aumentado tras MIO puede deberse a la curva de aprendizaje.

## Introduction

Oesophageal cancer is the sixth leading cause of cancer‐related death worldwide^1^. In early or locally advanced disease, surgery offers the best chance for

cure^2^. Oesophagectomy is an extensive operation followed by considerable risks of complications, mortality and poor health‐related quality of life^3^. With the aim of improving outcomes of oesophagectomy, the use of minimally invasive techniques has increased in recent years^4^.

Some studies^5^, ^6^, ^7^, ^8^, ^9^, ^10^, ^11^, ^12^, ^13^, ^14^ have suggested that minimally invasive oesophagectomy (MIO) reduces postoperative morbidity and mortality, shortens hospital stay, and improves patient satisfaction and health‐related quality of life compared with open oesophagectomy (OO), without compromising long‐term oncological outcomes. Three population‐based cohort studies^15^, ^16^, ^17^ have, however, found an increased risk of reoperation after MIO, possibly due to technical issues. A frequent complication of oesophagectomy is anastomotic stricture^18^, ^19^. Over 90 per cent of these strictures develop during the first year after surgery^18^. The incidence of stricture in an RCT^14^ was slightly higher after MIO (44 per cent) compared with that following OO (39 per cent), although the study was underpowered to test potential differences.

The aim of this study was to estimate the incidence of anastomotic stricture after oesophagectomy for cancer, and to compare the incidence of strictures requiring single or repeated dilatation between MIO and OO in a population‐based cohort from Finland and Sweden.

## Methods

All patients who had MIO or OO for cancer of the oesophagus in Finland or Sweden between 2007 and 2014 were included in the study. The two surgical approaches were compared regarding development of anastomotic stricture requiring one and repeated (3 or more) dilatations during the first year after surgery. The National Institute for Health and Welfare of Finland (permission numbers THL/143/5.05.00/2015 and THL/1349/5.05.00/2015) and the Regional Ethical Review Board in Stockholm, Sweden (DNR‐2015/1916‐31/1 and 2016/584‐32), approved the study.

All residents in both countries have unique and immutable ten‐ or 11‐digit personal identity codes, which allows for reliable patient identification from hospital records, administrative databases and national health data registries, and makes linkage of data between these databases possible. In this study, patients who underwent oesophagectomy for oesophageal cancer from 1 January 2007 to 31 December 2014 were identified from the Care Register for Healthcare in Finland and the Cancer Registry and Patient Registry in Sweden. Surgical codes JCC00, JCC10, JCC20, JCC30 and JCC96 were used. Endoscopic oesophageal dilatation (code JCA55) within 1 year of surgery was identified from the same registries. Surgical codes were recorded by the operating surgeon. Mortality data were linked for each patient individually from Statistics Finland and the Swedish Cause of Death Registry. The Finnish Hospital Discharge Registry and Care Register for Healthcare and the Swedish Patient Registry were also used to obtain data on patients' medical co‐morbidities, readmissions, length of hospital stay, performed procedures and discharge diagnoses.

Charlson co‐morbidity scores were calculated from diagnoses in the registries in 2004–2014, according to a validated algorithm^20^ and excluding oesophageal cancer. Gastric cancer was also excluded from the co‐morbidity index because the diagnoses of distal oesophageal and proximal gastric cancer can overlap in the diagnostic phase^21^. Standard procedures in Finland and Sweden during the study period were either Ivor Lewis oesophagectomy with intrathoracic anastomosis or a three‐phase procedure with cervical anastomosis for both MIO and OO^22^, ^23^.

The registries used have complete nationwide coverage, and reporting to the registries is compulsory in both countries. The coverage of the Finnish Hospital Discharge Registry and the Swedish Patient Registry are high because of the legislative obligation to report every inpatient treatment period and all outpatient contacts annually, linked to hospital funding. The Finnish Cancer Registry has a nationwide coverage over 99 per cent^24^, and the Swedish Cancer Registry has at least 98 per cent nationwide coverage for oesophageal cancer^21^. Both registers are considered reliable data sources^25^, ^26^.

### Statistical analysis

Baseline characteristics were analysed with the χ^2^ test or ANOVA tests, as appropriate. Multivariable Cox regression was used to calculate hazard ratios (HRs) with 95 per cent c.i. for oesophageal anastomotic strictures requiring single or repeated (3 or more) dilatations. OO was used as the reference group in the analyses. Two regression models were performed. The first had adjustment for seven variables considered to be potential confounding factors: age (continuous variable), sex (male or female), Charlson co‐morbidity score (0, 1 or 2 or more), histological tumour type (adenocarcinoma or squamous cell carcinoma), year of surgery (continuous), tumour stage (local or locally advanced) and country (Finland or Sweden). The second model included additional adjustment for hospital volume (low or high), length of hospital stay and readmission within 30 days after surgery, as proven proxies for complications^27^, ^28^. The definition of a high‐volume hospital was an annual number of resections above 20^28^. Owing to shortcomings in TNM data reported by the Finnish Cancer Registry, tumour staging was divided into local (T0–2 and N0) and locally advanced (T3–4 or N1 or above) disease. All analyses were conducted using the statistical software SAS® version 9.4 (SAS Institute, Cary, North Carolina, USA).

## Results

Between January 2007 and December 2014, 1669 patients underwent oesophagectomy for cancer in Finland or Sweden, including 239 (14·3 per cent) who had MIO and 1430 (85·7 per cent) who had OO. All were included in the present study (*Table* 1). The proportion of patients undergoing MIO increased over the study period. Resections were conducted in 35 hospitals, of which 11 performed both MIO and OO. Most MIOs were done in high‐volume hospitals (201 of 239, 84·1 per cent). The mean age at surgery was 64·8 years. Some 52·7 per cent of patients had a Charlson co‐morbidity score of zero, and adenocarcinoma was the most common histological type of cancer (74·4 per cent). Patients in the MIO group had less co‐morbidity and more locally advanced disease than those in the OO group. Otherwise there were no major baseline differences between the groups (*Table* 1).

**Table 1:** Clinical variables by surgical technique in patients who had oesophagectomy for oesophageal cancer in Finland and Sweden, 2007–2014

	All operations (*n* = 1669)	Open oesophagectomy (*n* = 1430)	Minimally invasive oesophagectomy (*n* = 239)
**Age (years)** *	64·8(9·3)	64·8(9·3)	64·8(9·5)
**Sex ratio (M : F)**	1332 : 337	1142 : 288	190 : 49
**Charlson co‐morbidity score**			
0	880 (52·7)	728 (50·9)	152 (63·6)
1	285 (17·1)	254 (17·8)	31 (13·0)
≥ 2	504 (30·2)	448 (31·3)	56 (23·4)
**Tumour histology** ‡			
Adenocarcinoma	1241 (74·4)	1053 (73·6)	188 (78·7)
Squamous cell carcinoma	384 (23·0)	343 (24·0)	41 (17·2)
**Stage**			
Local	413 (24·7)	368 (25·7)	45 (18·8)
Locally advanced	1056 (63·3)	891 (62·3)	165 (69·0)
Unknown	200 (12·0)	171 (12·0)	29 (12·1)
**Year of surgery**			
2007	185 (11·1)	184 (12·9)	1 (0·4)
2008	174 (10·4)	168 (11·7)	6 (2·5)
2009	196 (11·7)	182 (12·7)	14 (5·9)
2010	218 (13·1)	189 (13·2)	29 (12·1)
2011	211 (12·6)	187 (13·1)	24 (10·0)
2012	209 (12·5)	178 (12·4)	31 (13·0)
2013	239 (14·3)	197 (13·8)	42 (17·6)
2014	237 (14·2)	145 (10·1)	92 (38·5)
**Duration of hospital stay (days)** †	16 (12–24)	16 (12–24)	14 (12–21)
**Readmission within 30 days**	200 (12·0)	176 (12·3)	24 (10·0)

Values in parentheses are percentages unless indicated otherwise; values are

* mean(s.d.) and

† median (i.q.r.).

‡ In 44 patients no specific tumour histology was reported, or histology could not be determined (undefined carcinoma).

### Incidence of stricture

**Figure 1:**
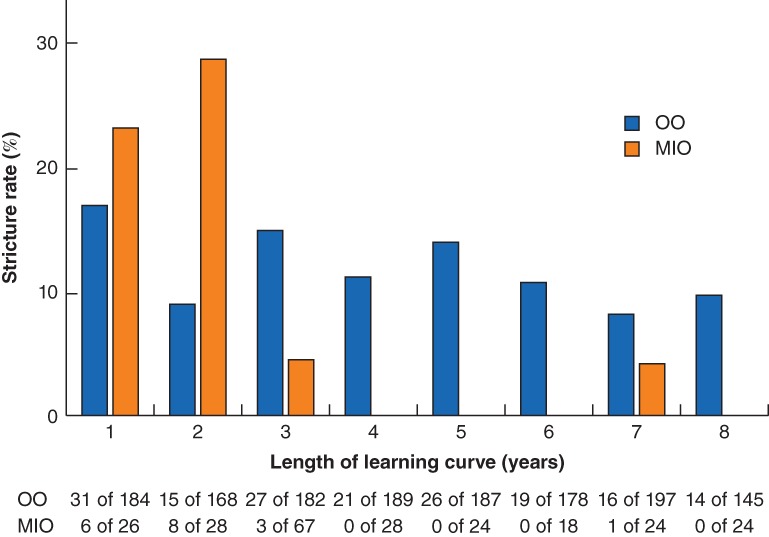
Effect of learning curve on anastomotic stricture rate The *x*‐axis presents the length of the learning curve in years, starting from 2007 (bars in year 1) for open oesophagectomy (OO) or from the introduction of minimally invasive oesophagectomy (MIO). For example, if MIO was started in 2012, this centre's stricture rate is included in bars 1–3 (2012–2014).

The overall incidence of strictures requiring one dilatation was 16·7 per cent (187 of 1119), and that for strictures needing three or more dilatations was 6·6 per cent (72 of 1084). The proportion of anastomotic strictures requiring at least a single dilatation in patients who reached 1‐year follow‐up was 14·3 per cent in the MIO group and 17·0 per cent in the OO group (*Table* 2). The majority of strictures (14 of 18, 78 per cent) occurred during the first 2 years after introduction of MIO, whereas the stricture rate was more stable after OO (*Fig*. 1). The corresponding proportions of patients requiring three or more dilatations were 8·9 per cent after MIO and 6·4 per cent after OO (*Table* 2). Most strictures occurred during the first 6 months after surgery: 94 per cent after MIO (17 of 18) and 87·0 per cent after OO (147 of 169).

**Table 2:** Incidence of anastomotic stricture requiring dilatation after oesophagectomy in Finland and Sweden, 2007–2014

Time frame of oesophageal dilatation	Open oesophagectomy	Minimally invasive oesophagectomy
0–3 months	93 of 1298 (7·2)	15 of 207 (7·2)
0–6 months	147 of 1191 (12·3)	17 of 181 (9·4)
0–12 months	169 of 993 (17·0)	18 of 126 (14·3)
≥ 3 dilatations, 0–12 months	61 of 960 (6·4)	11 of 124 (8·9)

Values in parentheses are percentages. Only patients who had dilatation, or who reached the set time point, are included in the total numbers of patients.

### Single dilatations of anastomotic strictures

The overall adjusted HR for one dilatation after MIO was 1·19 (95 per cent c.i. 0·66 to 2·12). In stratified analyses by country, Finland showed no association (adjusted HR 0·56, 0·11 to 2·94), whereas the corresponding data from Sweden showed an increased HR (adjusted HR 3·06, 1·59 to 5·89) (*Table* 3).

**Table 3:** Multivariable analysis of risk of single and repeated oesophageal dilatations within 1 year of minimally invasive and open oesophagectomy in Finland and Sweden, 2007–2014

		Hazard ratio	
	No. of patients	Open oesophagectomy	Minimally invasive oesophagectomy
**Single dilatation**			
Crude	1669	1·00 (reference)	0·67 (0·41, 1·09)
Adjustment 1*	1669	1·00 (reference)	1·09 (0·63, 1·89)
Adjustment 2†	1669	1·00 (reference)	1·19 (0·66, 2·12)
≥ 3 dilatations			
Crude	1669	1·00 (reference)	1·16 (0·61, 2·20)
Adjustment 1*	1669	1·00 (reference)	3·01 (1·39, 6·51)
Adjustment 2†	1669	1·00 (reference)	3·25 (1·43, 7·36)
**Stratified by country**			
Finland			
Single dilatation			
Crude	585	1·00 (reference)	0·17 (0·04, 0·70)
Adjustment 1*	585	1·00 (reference)	0·13 (0·03, 0·55)
Adjustment 2†	585	1·00 (reference)	0·56 (0·11, 2·94)
≥ 3 dilatations			
Crude	585	1·00 (reference)	1·00‡
Adjustment 1*	585	1·00 (reference)	1·00‡
Adjustment 2†	585	1·00 (reference)	1·00‡
**Sweden**			
Single dilatation			
Crude	1084	1·00 (reference)	1·90 (1·13, 3·20)
Adjustment 1*	1084	1·00 (reference)	3·19 (1·69, 6·03)
Adjustment 2†	1084	1·00 (reference)	3·06 (1·59, 5·89)
≥ 3 dilatations			
Crude	1084	1·00 (reference)	3·20 (1·67, 6·15)
Adjustment 1*	1084	1·00 (reference)	6·63 (2·95, 14·90)
Adjustment 2†	1084	1·00 (reference)	6·40 (2·73, 14·99)

Values in parentheses are 95 per cent confidence intervals.

* Adjusted for age, sex, Charlson Co‐morbidity Index, histological type, stage, year of surgery and country.

† Adjusted for age, sex, Charlson Co‐morbidity Index, histological type, stage, year of surgery, country, hospital volume, length of hospital stay and readmissions during 30 days.

‡ Confidence intervals cannot be estimated.

### Repeated dilatations of strictures up to 1 year after surgery

The overall adjusted HR for three or more dilatations after MIO was 3·25 (95 per cent c.i. 1·43 to 7·36). In stratified analyses by country, Finland showed no association, whereas in Sweden the adjusted HR was 6·40 (2·73 to 14·99) (*Table* 3).

### Secondary analysis of the risk of dilatation up to 1 year after surgery

In the fully adjusted model, age, sex, Charlson co‐morbidity score and year of surgery were not associated with an increased risk of single or repeated (3 or more) dilatations. The adjusted HR for squamous cell cancer histology was 1·49 (95 per cent c.i. 1·07 to 2·07) for single and 1·83 (1·10 to 3·03) for repeated dilatations. For locally advanced disease, the adjusted HR for single dilatation was 0·74 (0·54 to 1·00) and that for three or more dilatations was 0·45 (0·28 to 0·73).

In the analysis of previously identified proxies for complications, the overall adjusted HR for a single dilatation following oesophagectomy performed in a high‐volume centre was 0·88 (95 per cent c.i. 0·64 to 1·22), for patients with a longer hospital stay (per day) it was 1·00 (1·00 to 1·01), and for patients readmitted to hospital within 30 days of surgery it was 1·50 (1·00 to 2·27). Respective adjusted HRs for three of more dilatations were 0·84 (0·50 to 1·42), 1·01 (1·00 to 1·02) and 1·55 (0·80 to 3·02).

## Discussion

In this study, the rate of anastomotic stricture after oesophagectomy requiring an endoscopic dilatation was 16·7 per cent. The risk of anastomotic stricture requiring a single dilatation was similar after MIO and OO, but strictures requiring three or more dilatations appeared to be more common following MIO.

The main strengths of this study are the population‐based design with complete nationwide data from two Nordic countries, and the complete follow‐up of all patients. In addition, data regarding several confounding factors were available, and the analyses showed that these factors did influence risk estimates. Nevertheless, confounding by unmeasured factors might exist in this observational study. The lack of data on the location of the anastomosis is a concern, because cervical anastomosis is associated with a higher rate of leaks and strictures^19^, ^29^, ^30^. Differences between Finland and Sweden in minimally invasive technique existed, as most anastomoses were intrathoracic in Finland^23^ and cervical in Sweden^22^. Because the majority of oesophageal cancers are adenocarcinomas located in the distal oesophagus in both countries, the location of the anastomosis is often chosen by preference. To account for these differences, country was taken into account in stratified analyses. Owing to the lack of separate coding, it was not possible to differentiate totally minimally invasive surgery from hybrid procedures, although conversion rates have been relatively low in single‐centre reports from both countries^22^, ^23^, ^31^. Although it was not possible to adjust for complications or surgeon volume, hospital volume, length of stay and readmissions were used as proxies that can be considered reliable substitutes^27^, ^28^, ^32^.

Around 90 per cent of all strictures developed within the first 6 months after surgery, a similar rate to that found in one single‐centre study^18^. Previous case series^14^, ^19^, ^33^, ^34^ have reported wide ranges (6–44 per cent) of anastomotic stricture rates requiring dilatation, reflecting, in part, the rate of cervical anastomosis. Co‐morbidities, tumour location, chemoradiotherapy, anastomosis level and postoperative complications, especially anastomotic leak, have all been identified as significant risk factors for stricture development^19^, ^33^, ^35^, ^36^.

The only randomized study^14^ comparing MIO (59 patients) and OO (56 patients) showed no difference in stricture rate between the approaches (44 *versus* 39 per cent respectively), but was underpowered. In the present study, the risk of stricture requiring a single dilatation was similar with the two approaches, but the risk of stricture requiring three or more dilatations was higher after MIO. In previous population‐based studies assessing 30‐day outcomes after the two procedures, a higher reoperation rate was observed after MIO in three countries^15^, ^16^, ^17^, with a greater number of anastomosis leaks after MIO in the Netherlands^16^. It is possible that a learning curve relating to MIO affects these results^37^, ^38^. In the present study, a high stricture rate after the introduction of MIO programmes appears to imply a learning curve effect.

The present study also revealed a difference in the risk of anastomotic stricture between Finland and Sweden. MIO was not associated with strictures in Finland, but was a predisposing factor in Sweden for single and repeated dilatations. The two Nordic countries have similar demographics and healthcare systems, with balloon dilatation used to treat symptomatic strictures. The observed differences may be related to the differences in learning curves, location of anastomoses and technique^19^, ^34^. Based on previous studies^19^ and existing differences between countries in the present study, an intrathoracic anastomosis seems preferable to prevent anastomotic stricture.
